# The virome of HPV-positive tonsil squamous cell carcinoma and neck metastasis

**DOI:** 10.18632/oncotarget.27436

**Published:** 2020-01-21

**Authors:** Ryan M. Carey, Karthik Rajasekaran, Tyler Seckar, Xiang Lin, Zhi Wei, Charles C.L. Tong, Viran J. Ranasinghe, Jason G. Newman, Bert W. O'Malley, Gregory S. Weinstein, Michael D. Feldman, Erle S. Robertson

**Affiliations:** ^1^ Department of Otorhinolaryngology-Head and Neck Surgery, University of Pennsylvania, Perelman School of Medicine, Philadelphia, PA, USA; ^2^ Department of Computer Science, New Jersey Institute of Technology, Newark, NJ, USA; ^3^ Department of Pathology and Laboratory Medicine, University of Pennsylvania, Philadelphia, PA, USA; ^*^ Co-first authors

**Keywords:** HPV, cancer, virus, virome, oropharyngeal squamous cell carcinoma

## Abstract

Oropharyngeal squamous cell carcinoma (OPSCC) represents the most common HPV-related malignancy in the United States with increasing incidence. There is heterogeneity between the behavior and response to treatment of HPV-positive oropharyngeal squamous cell carcinoma that may be linked to the tumor virome. In this prospective study, a pan-pathogen microarray (PathoChip) was used to determine the virome of early stage, p16-positive OPSCC and neck metastasis treated with transoral robotic surgery (TORS) and neck dissection. The virome findings of primary tumors and neck lymph nodes were correlated with clinical data to determine if specific organisms were associated with clinical outcomes. A total of 114 patients were enrolled in the study. Double-stranded DNA viruses, specifically Papillomaviridae, showed the highest hybridization signal (viral copies) across all viral families in the primary and positive lymph node samples. High hybridization signals were also detected for signatures of Baculoviridae, Reoviridae, Siphoviridae, Myoviridae, and Polydnaviridae in most of the cancer specimens, including the lymph nodes without cancer present. Across all HPV signatures, HPV16 and 18 had the highest average hybridization signal index and prevalence. To our knowledge, this is the first study that has identified the viral signatures of OPSCC tumors. This will serve as a foundation for future research investigating the role of the virome in OPSCC. Further investigation into the OPSCC microbiome and its variations may allow for improved appreciation of the impact of microbial dysbiosis on risk stratification, oncologic outcomes, and treatment response which has been shown in other cancer sites.

## INTRODUCTION

Head and neck squamous cell carcinomas (HNSCCs), including oropharyngeal squamous cell carcinoma (OPSCC), are classically associated with risk factors such as tobacco and alcohol use [1]. However, human papillomavirus (HPV) has emerged as one of the primary causes of carcinogenesis for the majority of OPSCCs in North America and Europe [2]. Important clinical differences exist between HPV-positive and HPV-negative OPSCC which ultimately impact treatment decisions. HPV-positive OPSCCs have a higher incidence amongst younger patients with higher performance status, lower tobacco consumption, and higher socioeconomic status [3, 4]. Compared to HPV-negative OPSCCs, HPV-positive OPSCCs have an improved overall survival (OS) and disease-free survival (DFS) [5, 6], which is at least partially related to the increased sensitivity to chemotherapy and radiation therapy [7]. The survival advantage of HPV-positivity persists even after adjusting for confounders [8], suggesting a difference in the tumor biology between HPV-positive and -negative OPSCCs.

An improved understanding of prognostic factors and tumor biology is necessary to improve clinical outcomes and maximize quality of life in long-term survivors of HPV-positive OPSCC. Various studies have demonstrated that there are unique microbiomes associated with certain cancer types [9–14], including in oral and oropharyngeal squamous cell carcinomas [15]. These specific microbiomes may create a microenvironment conducive to oncogenesis, or it may be that the microenvironment in malignancy may promote specific microbiomes. Regardless, the interplay between the host and microbiome impacts the immune system on a local and systemic level that contributes to oncologic outcomes [16, 17]. Our previous work on the mixed group of p16-negative and -positive oral and oropharyngeal squamous cell carcinomas demonstrated that there were distinct viral and other microbial signature patterns associated with this type of malignancy [15]. Amongst the viruses, HPV, specifically HPV16 was the most detected molecular signature which is consistent with the fact that approximately 95% of HPV-positive OPSCCs are caused by HPV16 [18]. Additional viral signatures detected in our study included other HPVs, Herpesviridae, Poxviridae, Retroviridae and Polyomaviridae, and host integration hotspots were identified for HPV16 and JC polyomavirus [15].

In the present study, we utilized a pan-pathogen microarray (PathoChip [19]) to determine the viral microbiome of p16-positive, T1 or T2 tonsil squamous cell carcinomas with no positive nodes or a single <6 cm lymph node metastasis treated with transoral robotic surgery (TORS) and neck dissection. The virome findings were then correlated with clinical data to determine prognostic and diagnostic markers.

## RESULTS

### Clinical characteristics and outcomes

**Figure 1:**
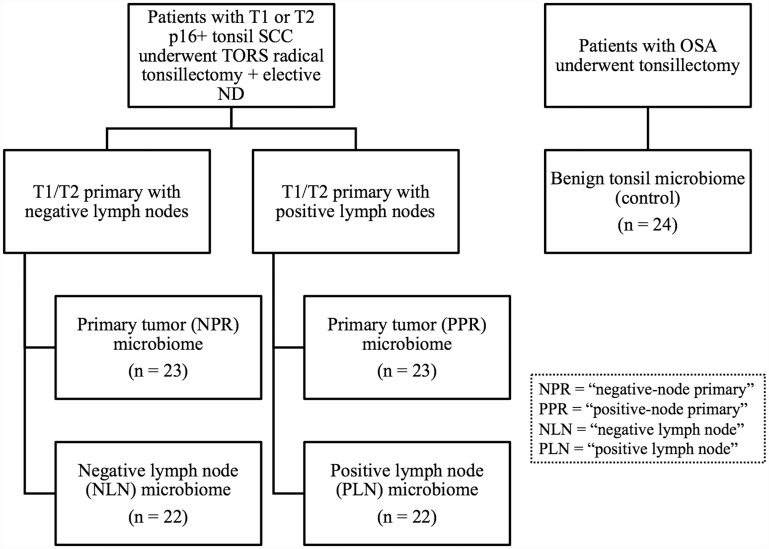
Study enrollment. Shown is the design of the study, including the three cohorts of patients and five cohorts of microbiome analyses. All patients with tonsil SCC underwent TORS radical tonsillectomy with elective neck dissection. Control patients underwent tonsillectomy alone as part of treatment for obstructive sleep apnea. SCC = squamous cell carcinoma; TORS = transoral robotic surgery; OSA = obstructive sleep apnea; ND = neck dissection.

After excluding inadequate tissue samples, a total of 114 patients were enrolled in the study. There were 23, 22, 23, 22, and 24 samples in the NPR (“negative-node primary”), NLN (“negative lymph node”), PPR (“positive-node primary”), PLN (“positive lymph node”), and control groups, respectively (Figure 1). Table 1 lists the demographic characteristics for patients included in the study. The difference in the proportions of patients who smoked from the negative-node SCC, positive-node SCC, and control groups was significant (71%, 74%, and 36%, *p* = 0.031). The average age for patients from the negative-node SCC, positive-node SCC, and control groups was significantly different (58.4, 57.0, and 46.4 years, *p* < 0.001).

**Table 1:** Demographic characteristics for all patients

	Tonsil SCC, negative nodes (*n* = 23)	Tonsil SCC, positive node (*n* = 23)	Control (*n* = 24)	*P*-value
**Age (mean (SD), years)**	58.4 ± 9.8	57.0 ± 9.8	46.4 ± 10.3	**<0.001**
**Sex (no. male, %)**	19 (79%)	20 (87%)	19 (76%)	0.903
**Race (no., %)**				
**White**	22 (92%)	21 (91%)	23 (96%)	0.779
**Black**	1 (4%)	2 (9%)	1 (4%)	0.779
**Hispanic**	1 (4%)	0 (0%)	1 (4%)	0.7
**Smoker^a^ (no., %)**	17 (71%)	17 (74%)	9 (36%)	**0.031**
**Diabetes (no., %)**	2 (8%)	3 (13%)	5 (25%)	0.398
**Hypertension (no., %)**	6 (25%)	8 (35%)	8 (32%)	0.727

SCC = squamous cell carcinoma.

^a^Smokers included current and former smokers.

Disease and treatment characteristics for patients with SCC are listed in Table 2. There were no statistically significant differences between pathologic factors between the negative-node and positive-node groups. Significantly more patients from the positive-node cohort received post-operative radiation therapy (70% vs 33%, *p* = 0.01). There were no statistically significant differences in the 2-year OS or DFS between the negative-node and positive-node groups.

**Table 2:** Disease characteristics for patients with SCC

	Tonsil SCC, negative nodes (*n* = 23)	Tonsil SCC, positive node (*n* = 23)	*P*-value
**Primary size (mean (SD), cm)**	2.37 ± 0.87	2.53 ± 0.77	0.51
**Positive margins (%)**	4%^a^	0%	0.32
**LVI (%)**	54%	74%	0.16
**PNI (%)**	0%	0%	0.60
**Node size (mean (SD), cm)**	n/a	3.42 ± 1.15	n/a
**ECS (%)**	n/a	0%	n/a
**Adjuvant treatment**			n/a
Chemotherapy	8%^b^	9%	0.97
Radiation therapy	33%	70%	**0.01**
**Outcomes**			
2-year overall survival^**c**^ (%)	90%	95%	0.58
2-year disease free survival^**c**^ (%)	81%	90%	0.97

SCC = squamous cell carcinoma; LVI = Lymphovascular invasion; PNI = Perineural invasion; ECS = Extracapsular extension.

^a^positive margin treated with chemoradiation including cetuximab.

^b^includes 1 patient who received cetuximab.

^c^patients with insufficient follow up were excluded from analysis.

### Viral signatures in p16-positive tonsil squamous cell carcinoma

**Figure 2:**
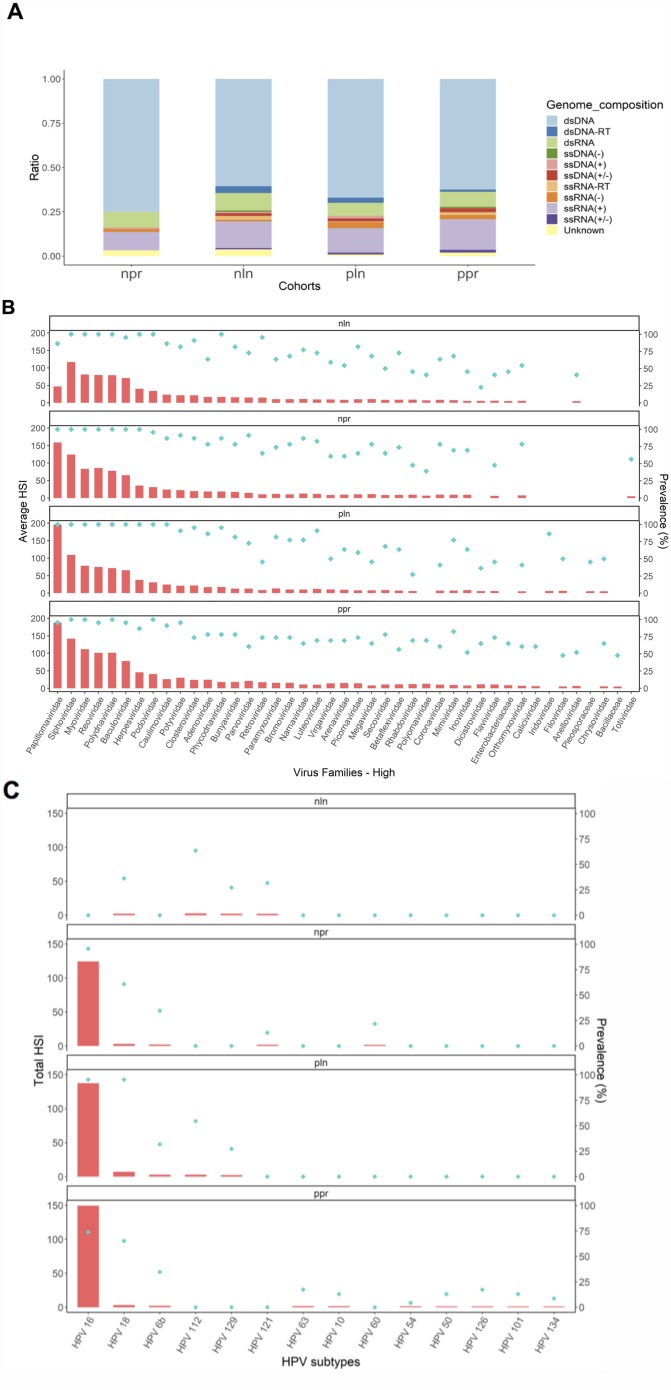
Viral signatures for tonsil cancer cohorts and controls by PathoChip screen. (**A**) Proportion of different viral signatures detected significantly in the tonsil cancer samples represented in bar graphs divided by viral types. (**B**) High level signatures of viral families for cancer cohorts, with the average hybridization signal as a bar graph and the prevalence as dots. (**C**) High level HPV signatures divided by subtype with the total hybridization signal as a bar graph and the prevalence as dots are shown in red and blue, respectively. NPR (“negative-node primary”), PPR (“positive-node primary”), NLN (“negative lymph node”), PLN (“positive lymph node”), HPV (human papillomavirus), *=*p* < 0.01, **= *p* < 0.001, ***= *p* < 0.0001.

Various DNA and RNA viral signatures were associated with the tonsil cancer groups (Figure 2). Double-stranded DNA viruses had the highest ratio amongst the viral signatures for all cohorts (*p* < 0.001), followed by single-stranded RNA (Figure 2A). There were 101 virus families with signatures. A hybridization signal index of > 5 was used to select virus families with relatively higher intensity signatures, of which there were 43. High hybridization signals were detected from Siphoviridae, Myoviridae, Reoviridae, Polydnaviridae, and Baculoviridae in most of the cancer specimens, including NLN (Figure 2B, Supplementary Table 1). Viral sequences belonging to the double-stranded DNA virus Papillomaviridae showed the highest hybridization signal across all viral families in the PPR, PLN, and NPR samples (Figure 2B, Supplementary Table 1). HPV detection by PathoChip was 95.7%, 100%, 100%, 86.4%, and 20.8% of PPR, NPR, PLN, NLN, and control specimens, respectively.

Fourteen HPV subtype signatures were detected with high hybridization signals amongst all cohorts, with the two high-risk subtypes HPV16 and HPV18, detected (Figure 2C). Across all HPV signatures, HPV16 had the highest average hybridization signal intensity and prevalence (Figure 2C). HPV16 was present in 73.9%, 95.7%, 95.5%, 0.00%, and 16.7% of PPR, NPR, PLN, NLN, and control specimens, respectively. Additionally, HPV18 maintained a high prevalence amongst the cohorts and was present in 65.2%, 69.6%, 95.5%, 45.5%, and 0.00% of PPR, NPR, PLN, NLN, and control specimens, respectively. Low-risk HPV6b had a prevalence of 34.8%, 34.8%, 31.8%, 0.00%, and 0.00% in PPR, NPR, PLN, NLN, and control specimens, respectively.

**Figure 3:**
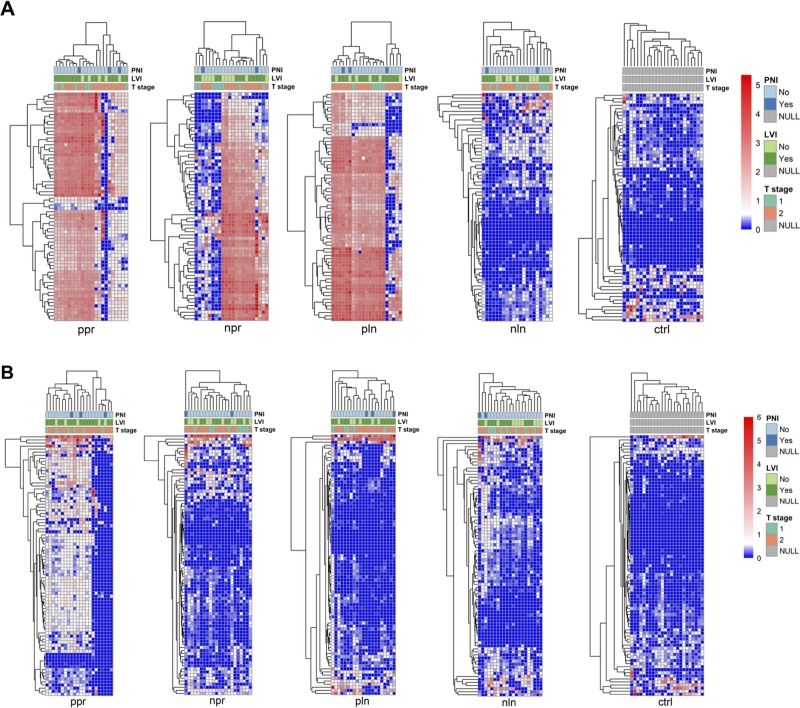
Hierarchical clustering of tonsil squamous cell carcinoma cohorts based on viral signature detection pattern with associated pathological features (perineural invasion (PNI), lymphovascular invasion (LVI), and tumor stage (T stage)). Hierarchical clustering for HPV16 (**A**) and HPV18 (**B**) viral probes are represented as heat maps for each cohort. Clustering was performed by R program using Euclidean distance, complete linkage and non-adjusted values. Clustering of the samples using NBClust software [Calinski and Harabasz index, Euclidean distance, complete linkage]. Chi-square test was applied and showed no significant differences of proportions of tumor stage (T1 versus T2), PNI, and LVI in different hierarchical clusters of cancer samples. NPR (“negative-node primary”), PPR (“positive-node primary”), NLN (“negative lymph node”), PLN (“positive lymph node”), CTRL (“control”), HPV (human papilloma virus), PNI (perineural invasion), LVI (lymphovascular invasion), T stage (tumor stage).

Due to the low number of deaths in each cohort, no significant probes were identified on Kaplan–Meier analysis of OS and DFS. Specifically, we could only perform survival analysis in the NPR group because of the limited number of deaths in the other cohorts. HPV16 probes had the highest hybridization signal indexes, prevalence, and number of probes within the NPR group. Comparisons of overall survival for high and low hybridization signal indexes for HPV16 probes in the NPR group were not significant (*p* = 0.082). There were no significant differences seen on evaluation of pathologic factors (tumor stage (T1 versus T2), PNI, LVI and tumors with a positive lymph node (NPR versus PPR)) in different hierarchical clusters of cancer samples for both HPV16 and HPV18 probes (Figure 3).

## DISCUSSION

We previously described the microbiome of oral and oropharyngeal squamous cell carcinomas [15]. In the current study, we evaluated the virome for a more homogeneous group of p16-positive, T1 or T2 tonsil squamous cell carcinomas with no positive lymph nodes or a single positive level 2 lymph node <6 cm, analyzing both the primary tumor and the lymph node specimens. To our knowledge, this is the first report of the virome of p16-positive tonsil squamous cell carcinoma. Our motivation for evaluating both pathologically positive and negative lymph node specimens in addition to primary tumors was to improve our understanding of the microenvironment necessary for or induced by regional metastasis. Furthermore, we attempted to determine clinical correlations with specific viral signatures.

In accordance with the NCCN treatment guidelines at the time of treatment for p16-positive T1-2, N0-N1 [20], patients in the study were treated with TORS primary resection and ipsilateral neck dissection followed by radiation or chemotherapy based on multidisciplinary review of pathology. We found no significant survival difference between N0 and N1 patients, which is consistent with prior studies [21, 22]. Haughey *et al*. demonstrated that increasing pathological N-classification was not associated with worse OS; in fact, N0 cases demonstrated worse OS than N1 in their analysis [21]. Based on prior studies of tobacco and HPV-associated OPSCC [6], the high prevalence of smoking (>70%) in our cohorts would be expected to negatively impact the clinical outcomes.

HPV infection is typically acquired through sexual contact, and the prevalence of oral HPV infection increases with the number of lifetime sexual partners and cigarettes smoked per day [23]. Prior larger studies have demonstrated that healthy controls have a prevalence of oral HPV infection of around 5% which is lower than our control group with 21%, possibly due to the higher rates of smoking in our cohort [23–25]. HPV16 is the most commonly detected carcinogenic HPV in oral samples of healthy patients [24], with prevalence around 1% [23–25]. We demonstrated a higher control prevalence of HPV16 (17%), but a low prevalence of the carcinogenic HPV18 (0%).

Though typically cleared by the immune system, in some individuals, the viral DNA integrates into the host genome [4] and can lead to OPSCC [2]. Persistent HPV16 DNA after treatment of HPV-positive OPSCC has been associated with worse survival [26]. Immunohistochemistry (IHC) for p16, a protein that increases with inhibition of pRb by E7, is currently the most widely utilized surrogate HPV biomarker [4, 27, 28], and is the method utilized at our institution for determining HPV-positivity in OPSCC. Interestingly, several studies have demonstrated some discordance between p16-positivity by IHC and the presence of HPV DNA via PCR [29–31]. Our results indicate a high concordance between p16-positivity and the presence of HPV signatures detected by PathoChip (100% prevalence in NPR and PLN and ~96% in PPR). Our previous work on primary oral and oropharyngeal squamous cell carcinoma demonstrated an average HPV16 prevalence of 98% [15], which was higher than the prevalence for the primary oropharyngeal tumor samples in the current study (~85%).

In contrast to our prior analysis of the mixed group of p16-negative and -positive oral and oropharyngeal squamous cell carcinomas [15], the current study contains a more homogeneous group of p16-positive OPSCC. Our previous study on p16-negative and -positive oral and oropharyngeal squamous cell carcinomas demonstrated high hybridization signals for Herpesviridae, Poxviridae, Retroviridae, Reoviridae, Orthomyxoviridae, and Polyomaviridae [15]. Similarly, the current study identified high hybridization signals for Baculoviridae, Reoviridae, Siphoviridae, Myoviridae, Polydnaviridae. Approximately 15% of human cancers are known to be caused by viruses [32], the majority of which are double-stranded DNA viruses [33]. The viruses with the highest hybridization signals in the cancer cohorts were predominantly double-stranded DNA viruses, with some exceptions including the double-stranded RNA virus Reoviridae. We suggest that the viruses identified in this study may represent signatures for p16-positive OPSCC.

The finding that Reoviridae had high hybridization signal in all cancer cohorts is particularly interesting because this virus is known to have strong specificity for cancer cells, particularly those with increased Ras activity [34–36]. Furthermore, Reovirus is known to have potent anti-tumor activity in many tumor models including HNSCC [37–39]. It is entirely possible that co-infection of Reovirus is increased in HPV-positive OPSCC compared to HPV-negative OPSCC, ultimately impacting the differences seen in survival. If true, Reovirus signatures may prove to be important biomarkers for OPSCC prognosis, helping to explain the heterogeneity in clinical responses and resistance to therapy in some tumors. Additional biomarkers for prognosis could aid in decisions regarding adjuvant therapy and treatment de-escalation to avoid morbidity. Regardless, this hypothesis is speculative and outside of the scope of the current study.

The viromes for the negative-node and positive-node primary tumors (NPR and PPR) had similar distributions of viral signatures. It remains to be determined if certain viruses impact the primary tumor’s ability to metastasize regionally. We did not identify any correlations between viral signatures and survival or patterns of failure; however, the number of recurrences and deaths in our study was likely too small to detect any differences. Similarly, the association between any viral probes and pathologic features will need to be explored with a larger scale study. Further work may determine that certain viruses induce alterations in gene expression to impact oncogenesis or cancer progression. These alterations may manifest in pathologic features such as tumor invasion of lymphatics and nerve or spread outside of lymph nodes; conversely, changes may not be appreciated microscopically and instead exist at the molecular level.

Although associations between specific viral signatures and p16-positive OPSCC disease characteristics remain to be determined, this study’s description of the OPSCC virome serves as a foundation for additional micobiome analyses. Several separate studies in the cancer literature suggest that host microbiome interactions effect oncologic outcomes [16, 17, 40, 41]. Specifically, it has been shown that the breast microbiome interacts with host cells to impact signaling pathways, ultimately modulating breast cancer growth and metastatic progression [40]. Within the pancreatic cancer literature, research has demonstrated that differences in microbiome diversity and the presence of specific microbial signatures predict long-term pancreatic adenocarcinoma survival [41]. Moreover, the tumor microbiome can be therapeutically altered in pancreatic adenocarcinoma with fecal microbiota transplantation, leading to changes in tumor growth and immune system response. Like the above-mentioned studies, additional work on the OPSCC microbiome may determine that cancer microbial dysbiosis impacts risk stratification and predicts oncologic outcomes and treatment response.

Important limitations exist with the current study. For example, the analysis and generalizability may be impacted by the control group having approximately double the prevalence of smoking compared to the estimated U. S. prevalence of 15.1% [42]. This increased smoking prevalence is likely related to the fact that patients with OSA have higher rates of smoking compared to those without OSA [43]. Additionally, the control group had about half the prevalence of smoking compared to the cancer group which may have impacted the analysis.

In summary, specific viruses, including HPV16, are known to impact the tumor biology and clinical behavior of OPSCCs. The virome of HPV-positive OPSCC primary tumors and neck lymph nodes include the virus families Papillomaviridae, Herpesviridae, Baculoviridae, Reoviridae, Siphoviridae, Myoviridae, and Polydnaviridae. Additional studies are necessary to determine if the identified viral signatures correlate with tumor behavior.

## MATERIALS AND METHODS

Experiments were performed in accordance with relevant guidelines and regulations with appropriate licensing and approvals by institutional committees at Perelman School of Medicine, University of Pennsylvania. The study was approved by the University of Pennsylvania institutional review board (protocol number 828709). The Cancer Center’s Clinical Trials Scientific Review and Monitoring Committee determined that this study met the requirements for review exemption.

### PathoChip design

The PathoChip Array design has been previously described [11, 19]. The array is comprised of 60,000 probe sets representing reference sequences of all known viruses, and human pathogenic bacteria, fungi, and parasites in Genbank. The PathoChip array contains probes which target genomic regions that are conserved between viruses in the same family and unique probes for each specific virus or microorganism. The arrays are manufactured as SurePrint glass slide microarrays (Agilent Technologies Inc.) with 8 replicate arrays per slide. Each probe is a 60 nucleotide (nt) DNA oligomer that targets multiple genomic regions of viruses, prokaryotic, and eukaryotic microorganisms. Accession annotations are available in the Gene Expression Omnibus (http://www.ncbi.nlm.nih.gov/geo/) [17].

### Study samples

Tonsil cancer and lymph node specimens were obtained from patients with p16 positive, T1 or T2 tonsil squamous cell carcinoma (staged with the American Joint Committee on Cancer (AJCC), 7th edition, TNM staging [44] and consistent with the current 8th edition [45]) treated with TORS and therapeutic or elective neck dissection. Only patients with no positive lymph nodes (N0) or a single positive level 2 lymph node less than 6 cm were included in the study (N1 by AJCC, 7th and 8th editions [44, 45]). All tumors were p16 positive determined by surgical biopsy or resection per the guidelines of the University of Pennsylvania Pathology Department and prior studies [46]. Specifically, samples are classified as p16 positive when p16 immunohistochemistry has greater than or equal to 70% of tumor nuclear and cytoplasmic staining.

Cases were divided into two groups based on the presence or absence of neck metastases after pathology review (Figure 1). For patients with no neck metastases, the primary tumor and matched negative neck lymph node specimens were denoted as NPR (“negative-node primary”) and NLN (“negative lymph node”), respectively. Alternatively, for patients with neck metastasis, the primary tumor and matched positive neck lymph node specimens were denoted as PPR (“positive-node primary”) and PLN (“positive lymph node”), respectively. The control group consisted of tonsil specimens from patients with obstructive sleep apnea (OSA) and no history of recurrent or chronic tonsillitis who underwent tonsillectomy as part of their treatment for OSA.

### Sample preparation and microarray processing

PathoChip screening utilized DNA and RNA extracted from formalin-fixed paraffin-embedded (FFPE) tissues as described previously [10, 19]. De-identified FFPE samples were received as 10 μm sections on non-charged glass slides. Tumor and control tissue were read by the pathologist at the Department of Pathology at the Hospital of the University of Pennsylvania, Philadelphia, PA. The samples were cut in a sterile fashion using a sterilized microtome.

The PathoChip screen procedure has been described in prior work [9–11, 15]. Briefly, DNA and RNA are extracted from the FFPE samples. Quality of extracted nucleic acids was determined by agarose gel electrophoresis and A260/280 measurements. Whole genome and transcriptome amplification (WTA) using the TransPlex Complete Whole Transcriptome Amplification Kit (Sigma-Aldrich, St. Louis, MO) was performed on extracted DNA and RNA samples (50 ng of DNA and 100 ng RNA) in accordance with manufacturer protocol. Reference human DNA and RNA was extracted from the human B cell line, BJAB (obtained from ATCC). The cell lines were cultured for less than 6 months and 15 ng of DNA and RNA were used for WTA. The human DNA and RNA served as a reference for determining cross-hybridization of probes to human sample amplified genome. After purifying the WTA products (PCR purification kit, Qiagen, Germantown, MD, USA), quality was checked using agarose gel electrophoresis and 1 μg of the amplified products from the cancer and control tissues were labelled with Cy3; the human reference was labelled with Cy5 (SureTag labeling kit, Agilent Technologies, Santa Clara, CA). All labeled specimens were purified and hybridized to the PathoChip microarrays, as previously described [11, 19]. For each PathoChip array, a Cy3 labelled sample and a Cy5 labelled reference was combined and hybridized together in constant rotation for 40 hr at 65°C. The slides were then washed and scanned for visualization using an Agilent SureScan G4900DA array scanner.

### Microarray data extraction and statistical analysis

The microarray data extraction and analyses techniques have been described previously [9–11, 19]. Briefly, Agilent Feature Extraction software was used for extraction of raw data from the microarray images, and the R program was used for data normalization and analyses [47]. The microarray screen data are available in Gene Expression Omnibus (Accession No. GSE111648). We calculated scale factors using the signals of green and red channels for human probes. Scale factors are the sum of green and sum of red signal ratios [∑(g)/∑(r)] of human probes. We used scale factors to obtain normalized signals for all other probes. For all probes except human probes, normalized signal is log2 transformed of green signals / scale factors modified red signals (log2 g – scale factor * log2 r). On the normalized signals, t-test is applied to select probes significantly present in cancer samples by comparing cancer samples versus controls and to select probes significantly present in the tonsil cancer samples versus the controls. The cut-off for significant detections in cancers versus the controls was log2 fold change > 1 and adjusted p value (with multiple testing corrections) < 0.05. Prevalence was calculated by counting the number of cancer cases with hybridization signal greater than the average signal of the negative control probes, and represented as a percentage.

Model-based analysis of tiling arrays (MAT) was used for detection of positive regions in the metagenome for each sample using the rMAT software [48]. This technique uses a sliding window to scroll through the entire metagenome of the array to detect positive hybridization signal (hot spots (highly expressed gene region) on the genome) [11, 19]. Analyses at the individual probe level (for both specific and conserved probes) and at the family level (taking into account all the probes per family) were performed.

The cancer samples were also subjected to unsupervised hierarchical clustering, based on the detection of microbial signatures in the samples (average hybridization signal per viral family or microbial genus), using the R program (Euclidean distance, complete linkage, non-adjusted values [47, 49], and the clusters were validated by Calinski and Harabasz index, which is implemented in R package as NbClust [50]. Calinski and Harabasz index is a cluster index that maximizes inter-cluster distances and minimizes intra-cluster distances. We calculated the possible cluster solution that would maximize the index values to achieve the best clustering of the data. The significant differences between the clusters observed by these methods were determined using two-sided t-test. The ANOVA test was carried out to find the common signatures significantly present in all the clusters.

### Clinical data and analysis of oncologic outcomes

Patients were followed prospectively following their initial diagnosis and treatment. Clinical information was obtained by an honest broker at the Hospital of the University of Pennsylvania who had access to the patient identifiers, but not the microbiome data. The clinical data was delivered to the statistician who matched the clinical information to the corresponding samples. The clinical information obtained for all samples included patient age, gender, ethnicity, smoking history, and presence of diabetes or hypertension. Additional data was collected for cancer patients including tumor size and stage, node size and stage, presence of lymphovascular invasion (LVI), perineural invasion (PNI), extracapsular spread (ECS), positive margins, and adjuvant treatment (radiation and/or chemotherapy). The outcome measures included 2-year OS and 2-year DFS. Chi-square test and t-test were applied to test whether there were any significant differences disease characteristics in the tonsil cancer patients with and without positive neck disease.

We performed Kaplan–Meier analysis for viral probes to identify correlations with OS and DFS. Chi-square test was applied to test whether there were any significant differences of proportions of pathologic factors in different hierarchical clusters of cancer samples.

## SUPPLEMENTARY MATERIALS





## References

[1] Timbang MR, Sim MW, Bewley AF, Farwell DG, Mantravadi A, Moore MG HPV-related oropharyngeal cancer: a review on burden of the disease and opportunities for prevention and early detection. Hum Vaccin Immunother. 2019; 15:1920–1928. 10.1080/21645515.2019.1600985. 31050595 PMC6746516

[2] Gooi Z, Chan JY, Fakhry C The epidemiology of the human papillomavirus related to oropharyngeal head and neck cancer. Laryngoscope. 2016; 126:894–900. 10.1002/lary.25767. 26845348

[3] Ferlay J, Soerjomataram I, Dikshit R, Eser S, Mathers C, Rebelo M, Parkin DM, Forman D, Bray F Cancer incidence and mortality worldwide: sources, methods and major patterns in GLOBOCAN 2012. Int J Cancer. 2015; 136:E359–E386. 10.1002/ijc.29210. 25220842

[4] Chai RC, Lambie D, Verma M, Punyadeera C Current trends in the etiology and diagnosis of HPV-related head and neck cancers. Cancer Med. 2015; 4:596–607. 10.1002/cam4.424. 25644715 PMC4402074

[5] Weinberger PM, Yu Z, Haffty BG, Kowalski D, Harigopal M, Brandsma J, Sasaki C, Joe J, Camp RL, Rimm DL, Psyrri A Molecular classification identifies a subset of human papillomavirus–associated oropharyngeal cancers with favorable prognosis. J Clin Oncol. 2006; 24:736–747. 10.1200/JCO.2004.00.3335. 16401683

[6] Ang KK, Harris J, Wheeler R, Weber R, Rosenthal DI, Nguyen-Tan PF, Westra WH, Chung CH, Jordan RC, Lu C, Kim H, Axelrod R, Silverman CC, et al Human papillomavirus and survival of patients with oropharyngeal cancer. N Engl J Med. 2010; 363:24–35. 10.1056/NEJMoa0912217. 20530316 PMC2943767

[7] Fakhry C, Westra WH, Li S, Cmelak A, Ridge JA, Pinto H, Forastiere A, Gillison ML Improved survival of patients with human papillomavirus-positive head and neck squamous cell carcinoma in a prospective clinical trial. J Natl Cancer Inst. 2008; 100:261–269. 10.1093/jnci/djn011. 18270337

[8] Benson E, Li R, Eisele D, Fakhry C The clinical impact of HPV tumor status upon head and neck squamous cell carcinomas. Oral Oncol. 2014; 50:565–574. 10.1016/j.oraloncology.2013.09.008. 24134947 PMC4391706

[9] Banerjee S, Tian T, Wei Z, Shih N, Feldman MD, Alwine JC, Coukos G, Robertson ES The ovarian cancer oncobiome. Oncotarget. 2017; 8:36225–36245. 10.18632/oncotarget.16717. 28410234 PMC5482651

[10] Banerjee S, Tian T, Wei Z, Shih N, Feldman MD, Peck KN, DeMichele AM, Alwine JC, Robertson ES Distinct Microbial Signatures Associated With Different Breast Cancer Types. Front Microbiol. 2018; 9:951. 10.3389/fmicb.2018.00951. 29867857 PMC5962706

[11] Banerjee S, Wei Z, Tan F, Peck KN, Shih N, Feldman M, Rebbeck TR, Alwine JC, Robertson ES Distinct microbiological signatures associated with triple negative breast cancer. Sci Rep. 2015; 5:15162. 10.1038/srep15162. 26469225 PMC4606812

[12] Castano-Rodriguez N, Goh KL, Fock KM, Mitchell HM, Kaakoush NO Dysbiosis of the microbiome in gastric carcinogenesis. Sci Rep. 2017; 7:15957. 10.1038/s41598-017-16289-2. 29162924 PMC5698432

[13] Sheflin AM, Whitney AK, Weir TL Cancer-promoting effects of microbial dysbiosis. Curr Oncol Rep. 2014; 16:406. 10.1007/s11912-014-0406-0. 25123079 PMC4180221

[14] Xuan C, Shamonki JM, Chung A, Dinome ML, Chung M, Sieling PA, Lee DJ Microbial dysbiosis is associated with human breast cancer. PLoS One. 2014; 9:e83744. 10.1371/journal.pone.0083744. 24421902 PMC3885448

[15] Banerjee S, Tian T, Wei Z, Peck KN, Shih N, Chalian AA, O’Malley BW, Weinstein GS, Feldman MD, Alwine J, Robertson ES Microbial Signatures Associated with Oropharyngeal and Oral Squamous Cell Carcinomas. Sci Rep. 2017; 7:4036. 10.1038/s41598-017-03466-6. 28642609 PMC5481414

[16] Gopalakrishnan V, Helmink BA, Spencer CN, Reuben A, Wargo JA The Influence of the Gut Microbiome on Cancer, Immunity, and Cancer Immunotherapy. Cancer Cell. 2018; 33:570–580. 10.1016/j.ccell.2018.03.015. 29634945 PMC6529202

[17] Zitvogel L, Ma Y, Raoult D, Kroemer G, Gajewski TF The microbiome in cancer immunotherapy: Diagnostic tools and therapeutic strategies. Science. 2018; 359:1366–1370. 10.1126/science.aar6918. 29567708

[18] Dayyani F, Etzel CJ, Liu M, Ho CH, Lippman SM, Tsao AS Meta-analysis of the impact of human papillomavirus (HPV) on cancer risk and overall survival in head and neck squamous cell carcinomas (HNSCC). Head Neck Oncol. 2010; 2:15. 10.1186/1758-3284-2-15. 20587061 PMC2908081

[19] Baldwin DA, Feldman M, Alwine JC, Robertson ES Metagenomic assay for identification of microbial pathogens in tumor tissues. MBio. 2014; 5:e01714–e14. 10.1128/mBio.01714-14. 25227467 PMC4172075

[20] Network NCC Head and Neck Cancers (Version 2.2019). 2019.

[21] Haughey BH, Sinha P, Kallogjeri D, Goldberg RL, Lewis JS Jr, Piccirillo JF, Jackson RS, Moore EJ, Brandwein-Gensler M, Magnuson SJ, Carroll WR, Jones TM, Wilkie MD, et al Pathology-based staging for HPV-positive squamous carcinoma of the oropharynx. Oral Oncol. 2016; 62:11–19. 10.1016/j.oraloncology.2016.09.004. 27865363 PMC5523818

[22] Cramer JD, Dundar Y, Hotaling J, Raza SN, Lin HS Development and Assessment of a Novel Composite Pathologic Risk Stratification for Surgically Resected Human Papillomavirus-Associated Oropharyngeal Cancer. JAMA Otolaryngol Head Neck Surg. 2019; 145:1105. 10.1001/jamaoto.2019.0820. 31042786 PMC6495356

[23] Gillison ML, Broutian T, Pickard RK, Tong ZY, Xiao W, Kahle L, Graubard BI, Chaturvedi AK Prevalence of oral HPV infection in the United States, 2009-2010. JAMA. 2012; 307:693–703. 10.1001/jama.2012.101. 22282321 PMC5790188

[24] Kreimer AR, Villa A, Nyitray AG, Abrahamsen M, Papenfuss M, Smith D, Hildesheim A, Villa LL, Lazcano-Ponce E, Giuliano AR The epidemiology of oral HPV infection among a multinational sample of healthy men. Cancer Epidemiol Biomarkers Prev. 2011; 20:172–182. 10.1158/1055-9965.EPI-10-0682. 21148755 PMC3027138

[25] Kreimer AR, Bhatia RK, Messeguer AL, Gonzalez P, Herrero R, Giuliano AR Oral human papillomavirus in healthy individuals: a systematic review of the literature. Sex Transm Dis. 2010; 37:386–391. 20081557 10.1097/OLQ.0b013e3181c94a3b

[26] Rettig EM, Wentz A, Posner MR, Gross ND, Haddad RI, Gillison ML, Fakhry C, Quon H, Sikora AG, Stott WJ, Lorch JH, Gourin CG, Guo Y, et al Prognostic Implication of Persistent Human Papillomavirus Type 16 DNA Detection in Oral Rinses for Human Papillomavirus-Related Oropharyngeal Carcinoma. JAMA Oncol. 2015; 1:907–915. 10.1001/jamaoncol.2015.2524. 26226294 PMC7286348

[27] Plummer M, de Martel C, Vignat J, Ferlay J, Bray F, Franceschi S Global burden of cancers attributable to infections in 2012: a synthetic analysis. Lancet Glob Health. 2016; 4:e609–e616. 10.1016/S2214-109X(16)30143-7. 27470177

[28] Mendelsohn AH, Lai CK, Shintaku IP, Elashoff DA, Dubinett SM, Abemayor E, St John MA Histopathologic findings of HPV and p16 positive HNSCC. Laryngoscope. 2010; 120:1788–1794. 10.1002/lary.21044. 20803740

[29] Hoffmann M, Tribius S, Quabius ES, Henry H, Pfannenschmidt S, Burkhardt C, Gorogh T, Halec G, Hoffmann AS, Kahn T, Rocken C, Haag J, Waterboer T, et al HPV DNA, E6*I-mRNA expression and p16INK4A immunohistochemistry in head and neck cancer - how valid is p16INK4A as surrogate marker? Cancer Lett. 2012; 323:88–96. 10.1016/j.canlet.2012.03.033. 22484467

[30] Lewis JS Jr, Thorstad WL, Chernock RD, Haughey BH, Yip JH, Zhang Q, El-Mofty SK p16 positive oropharyngeal squamous cell carcinoma:an entity with a favorable prognosis regardless of tumor HPV status. Am J Surg Pathol. 2010; 34:1088–1096. 10.1097/PAS.0b013e3181e84652. 20588174 PMC3873742

[31] Robinson M, Sloan P, Shaw R Refining the diagnosis of oropharyngeal squamous cell carcinoma using human papillomavirus testing. Oral Oncol. 2010; 46:492–496. 10.1016/j.oraloncology.2010.02.013. 20227331

[32] Rampias T, Sasaki C, Weinberger P, Psyrri A E6 and e7 gene silencing and transformed phenotype of human papillomavirus 16-positive oropharyngeal cancer cells. J Natl Cancer Inst. 2009; 101:412–423. 10.1093/jnci/djp017. 19276448

[33] McBride AA The Promise of Proteomics in the Study of Oncogenic Viruses. Mol Cell Proteomics. 2017; 16:S65–S74. 10.1074/mcp.O116.065201. 28104704 PMC5393395

[34] Shmulevitz M, Lee PW Exploring host factors that impact reovirus replication, dissemination, and reovirus-induced cell death in cancer versus normal cells in culture. Methods Mol Biol. 2012; 797:163–176. 10.1007/978-1-61779-340-0_12. 21948476

[35] Marcato P, Shmulevitz M, Pan D, Stoltz D, Lee PW Ras transformation mediates reovirus oncolysis by enhancing virus uncoating, particle infectivity, and apoptosis-dependent release. Mol Ther. 2007; 15:1522–1530. 10.1038/sj.mt.6300179. 17457318

[36] Shmulevitz M, Marcato P, Lee PW Activated Ras signaling significantly enhances reovirus replication and spread. Cancer Gene Ther. 2010; 17:69–70. 10.1038/cgt.2009.46. 19590528

[37] Ikeda Y, Nishimura G, Yanoma S, Kubota A, Furukawa M, Tsukuda M Reovirus oncolysis in human head and neck squamous carcinoma cells. Auris Nasus Larynx. 2004; 31:407–412. 10.1016/S0385-8146(04)00111-7. 15571915

[38] Cooper T, Biron VL, Fast D, Tam R, Carey T, Shmulevitz M, Seikaly H Oncolytic activity of reovirus in HPV positive and negative head and neck squamous cell carcinoma. J Otolaryngol Head Neck Surg. 2015; 44:8. 10.1186/s40463-015-0062-x. 25890191 PMC4348167

[39] Pan D, Pan LZ, Hill R, Marcato P, Shmulevitz M, Vassilev LT, Lee PW Stabilisation of p53 enhances reovirus-induced apoptosis and virus spread through p53-dependent NF-kappaB activation. Br J Cancer. 2011; 105:1012–1022. 10.1038/bjc.2011.325. 21863032 PMC3185941

[40] Parida S, Sharma D The power of small changes: Comprehensive analyses of microbial dysbiosis in breast cancer. Biochim Biophys Acta Rev Cancer. 2019; 1871:392–405. 10.1016/j.bbcan.2019.04.001. 30981803 PMC8769497

[41] Riquelme E, Zhang Y, Zhang L, Montiel M, Zoltan M, Dong W, Quesada P, Sahin I, Chandra V, San Lucas A, Scheet P, Xu H, Hanash SM, et al Tumor Microbiome Diversity and Composition Influence Pancreatic Cancer Outcomes. Cell. 2019; 178:795–806.e12. 10.1016/j.cell.2019.07.008. 31398337 PMC7288240

[42] Jamal A, King BA, Neff LJ, Whitmill J, Babb SD, Graffunder CM Current Cigarette Smoking Among Adults - United States, 2005-2015. MMWR Morb Mortal Wkly Rep. 2016; 65:1205–1211. 10.15585/mmwr.mm6544a2. 27832052

[43] Zhu HM, Yi HL, Guan J, Xu HJ, Liu SR, Zou JY, Chen R [Relationship between smoking and the severity of OSA]. Lin Chung Er Bi Yan Hou Tou Jing Wai Ke Za Zhi. 2019; 33:862–5; 9. 10.13201/j.issn.1001-1781.2019.09.016. 31446706

[44] Edge SB, Compton CC The American Joint Committee on Cancer: the 7th edition of the AJCC cancer staging manual and the future of TNM. Ann Surg Oncol. 2010; 17:1471–4. 10.1245/s10434-010-0985-4. 20180029

[45] Amin MB, American Joint Committee on Cancer American Cancer Society AJCC cancer staging manual. (Chicago IL: American Joint Committee on Cancer, Springer). 2017.

[46] Begum S, Gillison ML, Ansari-Lari MA, Shah K, Westra WH Detection of human papillomavirus in cervical lymph nodes: a highly effective strategy for localizing site of tumor origin. Clin Cancer Res. 2003; 9:6469–6475. 14695150

[47] Team RC R: A Language and Environment for Statistical Computing Vienna, Austria: R Foundation for Statistical Computing; 2019.

[48] Droit A, Cheung C, Gottardo R rMAT–an R/Bioconductor package for analyzing ChIP-chip experiments. Bioinformatics. 2010; 26:678–679. 10.1093/bioinformatics/btq023. 20089513

[49] Kolde R. Pheatmap: Pretty Heatmaps. R Package Version 1.0.2. 2015.

[50] Charrad M, Ghazzali N, Boiteau V, Nifnafs A NbClust: an R package for determining the relevant number of clusters in a data set. J Stat Softw. 2014; 61:1–36. 10.18637/jss.v061.i06.

