# Supplementary material for: The virome of HPV-positive tonsil squamous cell carcinoma and neck metastasis
Source: Oncotarget. 2020 Jan 21;11(3):282–93. doi: 10.18632/oncotarget.27436 (PMC6980631; doi:10.18632/oncotarget.27436)
Supplement: Supplementary file 1 [file oncotarget-11-282-s001.pdf]

The virome of HPV-positive tonsil squamous cell carcinoma and neck metastasis

SUPPLEMENTARY MATERIALS

Supplementary Table 1. Significant detection of viral probes in cancer compared to non-matched controls. Hybridization signal intensity was calculated and compared between cancer and non-matched controls, and significance (*p*-value < 0.05) was calculated using one sided t-tests. Note that not all probes are shown. See Supplementary Table 1
